# Hypersensitivity Pneumonitis: Challenges of a Complex Disease

**DOI:** 10.1155/2024/4919951

**Published:** 2024-01-18

**Authors:** Diana Calaras, Aliona David, Eirini Vasarmidi, Katerina Antoniou, Alexandru Corlateanu

**Affiliations:** ^1^Department of Pulmonology and Allergology, State University of Medicine and Pharmacy “Nicolae Testemitanu”, Chisinau, Moldova; ^2^Outpatient Department, Institute of Phtisiopneumology “Chiril Draganiuc”, Chisinau, Moldova; ^3^Department of Respiratory Medicine, Laboratory of Molecular and Cellular Pulmonology, School of Medicine, University of Crete, Heraklion, Greece

## Abstract

Hypersensitivity pneumonitis (HP) is a complex interstitial lung disease caused by chronic inhalation of a wide variety of antigens in susceptible and sensitized individuals, commonly associated with an occupational exposure. An impressive number of inciting antigens causing hypersensitivity pneumonitis have been found to cover a wide range of occupations. As working practices have changed over time, especially in industrialized countries, new names for occupational HP have emerged. This review emphasizes the main diagnostic issues arising from the high variability of clinical presentation and the broad spectrum of causal antigens. Furthermore, it provides an overview of current methods to unveil possible causes of hypersensitivity pneumonitis, highlights HP's current diagnostic and treatment challenges and the remaining areas of uncertainty, and presents prevention strategies.

## 1. Introduction

Hypersensitivity pneumonitis (HP) is a complex interstitial lung disease caused by an immune-mediated inflammation of the lungs driven by chronic inhalation of a wide variety of antigens in susceptible and sensitized individuals, usually found in adults and commonly associated with an occupational setting [1].

Over time, the concept of hypersensitivity pneumonitis, also known as “allergic extrinsic alveolitis,” has evolved. It was first described 100 years ago in farmers exposed to moldy hay, termed farmer's lung [2]. Nowadays, more than 200 inciting antigens causing HP have been found to cover a wide range of occupations [3], bird fancier's lung being the most common [4]. As working practices have changed over time, especially in industrialized countries, occupational HP names, such as coffee worker's lung, paprika splitter's lung, and malt workers' lung, are less frequently reported, and others, such as HP induced by metalworking fluids, have emerged [5].

Depending on the inciting antigen type and length of antigen exposure in an individual with a genetic predisposition, the disease can have a heterogeneous clinical presentation that varies from asymptomatic sensitization to a certain antigen in exposed individuals to progressive lung fibrosis [6] and can be expressed as a wide variety of imaging patterns, morphological appearance, and outcome [1, 7]. This disease heterogeneity implies great uncertainty in establishing a diagnosis in the absence of a gold-standard diagnostic test. In the past years, progress has been made regarding the classification of HP, diagnostic evaluation, and management algorithm in patients with suspected HP, with many clinical challenges left over despite the recent publication of two international consensus guidelines [6, 8]. Currently, the diagnosis is a matter of either the clinician's judgment or a multidisciplinary team (MDT) discussion, based on a combination of a thorough history, clinical and imaging data, bronchoalveolar lavage (BAL) pattern, and serum immunological and histological findings [9].

HP prognosis can be substantially improved by antigen avoidance [10]. Therefore, identifying the common sources of occupational exposure will ensure earlier avoidance and stop further exposure to offending antigens.

Being considered mostly as an inflammatory disease, immunosuppressive drugs such as corticosteroids and other corticosteroid-sparing agents have an important role, while antifibrotic agents show promising results in progressive fibrotic lung disease.

This review presents an update regarding HP's epidemiology, antigen diversity, diagnostic challenges, and management strategies.

## 2. Epidemiology

Given the wide variability of clinical presentation and the lack of consensus over a definition for HP, the exact incidence and prevalence of the disease in the general population remain unknown. Extrapolating the results of several population-based studies to the general population, there is an estimated incidence of 0.13–1.94 cases per 100 000 and a prevalence of 0.45–2.71 cases per 100 000 [11–13] and tends to increase with age to 11.2 cases per 100 000 in patients older than 65 [11].

HP is a rare disease affecting mainly adults with a mean age of 50–60 years [14], involving men and women almost equally [13, 15], with some local variations found in a UK epidemiological study, which reported men being affected in an occupational setting four times more frequently than women [14].

Significant variations in the prevalence of occupational HP are observed from one country to another due to geographical, climate, and seasonal differences, type and quantity of antigens, level of industrialization, agricultural techniques, and other features of the professional environment. Therefore, the estimated burden of occupational HP ranges from 0% to 81.3% of individuals with high-risk occupations [16].

## 3. Antigens and Occupational Sources of Exposure

The antigens triggering HP are either of organic origin (animal or plant proteins, bacteria, and fungi) or inorganic agents such as metals and chemicals [17]. Given the widespread persistence of these antigens, HP can commence in any environment: workplace, home, and recreational. Nevertheless, every 5^th^ case of HP has an occupational origin; individuals working in agriculture, the food industry, those exposed to metalworking fluids, processing wood, working in construction, and textile manufacturing are at the highest risk (Table 1) [14].

About 30% of all HP cases can be attributed to bird exposure (pigeons, parrots and canaries, duck, goose, and dawn) and bird-derived products like feathers and droppings [4], causing bird fancier's lung. Farming represents the second most common occupation, with a vast spectrum of workplace exposures. Decaying vegetation, silage, fruits, vegetables, seeds, soil and organic fertilizers (organic waste and compost), and greenhouses are common sources of bacterial and fungal antigens for HP.

Due to evolving workplace practices, farmer's lung has become less frequent. At the same time, exposures to various species of bacteria, mycobacteria, and fungi from contaminated aerosolized water have been increasingly reported in water-related pursuits such as machine operators, workers with ultrasonic humidifiers, steam irons, air conditioners, hot tubs, swimming pools, hydroponic cultivation, and windpipe musical instruments [5, 18].

Individuals working in construction and manufacturing are frequently exposed to isocyanates, the small-molecular-weight substances derived from plastics and polyurethanes, usually found in insulating spray foams, varnishes, paints, and coatings that are recognized as the leading cause of occupational asthma in industrialized countries [19]. The isocyanate compounds can also trigger non-IgE-mediated delayed hypersensitivity reactions, such as HP. Some occupational studies suggest that isocyanate-induced HP prevalence could reach up to 27% in exposed workers [20].

Besides the workplace, the home environment can be an important source of sensitization, coming from feather duvets, carpets, moldy dwellings and bathrooms, and car air conditioning with the most frequently involved causal agents including *Aspergillus* spp.*, Penicillium* sp.*, Wallemia sebi, Botrytis cinerea, Trichoderma pseudokoningii, Cephalotrichum* sp., and *Thermoactinomyces vulgaris* [21].

## 4. Clinical Presentation

Conventionally, HP was classified as acute, subacute, and chronic forms based on the duration of symptoms. However, the criteria for defining these forms were very equivocal, leading to an overlap of the subacute form with the acute and chronic. Moreover, this classification failed to show an association with prognosis. Since the outcome of HP is directly determined by the presence of fibrosis, two recent guidelines [6, 8] have taken up the two cluster classification concepts that divided HP into nonfibrotic and fibrotic, two phenotypes that vary from pure inflammation to a mix of inflammation with fibrosis of a various degree of extent. However, Costabel et al. suggested preserving acute HP as an entity due to its utility in characterizing outbreaks of HP observed especially in an occupational environment [22].

Clinical presentation is heterogeneous and mostly nonspecific, varying from productive cough, dyspnea, and fatigue often associated with an intermittent flu-like syndrome to insidious disease with almost no symptoms for weeks and months. In high-grade exposures to the offending antigens, symptoms commonly start after 6–8 hours of exposure, that is usually at the end of the working day, and resolve after 24–48 hours of exposure discontinuance, meaning that patient may experience symptoms during working days and improve in the weekend off work. In contrast, low-grade exposure may not have very expressive clinical symptoms, and the correlation with the working shift or days could be absent [5]. A summary of the clinical presentation of the two phenotypes of HP is presented in Table 2.

## 5. Diagnostic Criteria

There is great uncertainty in establishing the diagnosis of HP since currently there is no gold-standard diagnostic test. The confusion grows even after two recent guidelines [6, 8] could not agree upon the diagnostic criteria (Table 3). Thus, the diagnosis of HP resembles a puzzle that can be solved by matching multiple pieces: (a) clinical features (inspiratory crackles, squeaks), (b) exposure identification, that includes either a positive exposure history and/or the presence of serum IgG against potential antigens (Table 1), (c) suggestive HRCT imaging, (d) lung function, and (e) BAL lymphocytosis [6]. Both guidelines conceded that suspected cases should be discussed in a multidisciplinary team (MDT) comprised of clinicians, radiologists, and occupational physicians, when necessary, that would decide the need for surgical lung biopsy.

### 5.1. Exposure Assessment

#### 5.1.1. Occupational History

**Figure 1:**
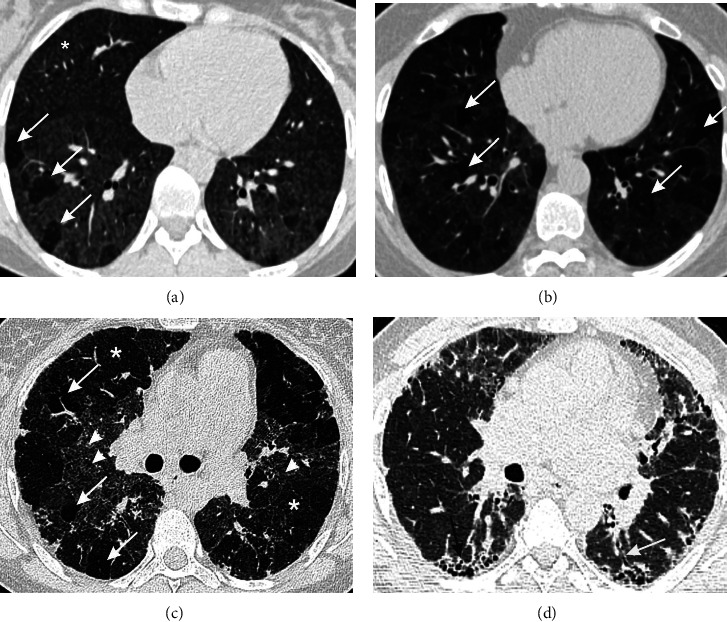
Imaging patterns in HP. (a) Axial section of chest high-resolution computer tomography (HRCT) showing ill-defined centrilobular nodules (white asterisk) and areas of air-trapping (arrows) suggesting small airway involvement. (b) Ground-glass opacities and mosaic attenuation (ground glass alternating with air-trapping (arrows)) suggestive of predominant interstitial infiltration. (c) The “three density sign” comprised of lung lobules with normal density (white asterisk), lobules with ground-glass attenuation (arrowheads), and lobules with decreased density due to air-trapping (arrows). (d) Fibrotic HP presented by areas of ground glass accompanied by traction bronchiectasis (black arrow) and honeycombing (white arrow).

Diagnosing HP may be challenging since clinical signs, and imaging data may overlap with other ILDs. This is why HP should be considered a potential diagnosis in any ILD case [6]. An obvious exposure history associated with a suggestive imaging pattern may be acceptable without serological or histopathological confirmation. Moreover, a strong exposure history in a patient with usual interstitial pneumonia (UIP)-like pattern might make the difference between idiopathic pulmonary fibrosis (IPF) and HP [23]. Thus, exposure/occupational history should be a mandatory diagnostic tool in the diagnostic algorithm of a newly diagnosed patient with ILD (Figure 1).

It has been established that HP with unknown exposure is associated with poor prognosis [10], and unfortunately, in nearly half of HP cases, the offending antigens cannot be recognized [24]. Several measures could contribute to increasing the chances of identifying the exposures:

- Collecting a thorough environmental history [25], which could be facilitated by using relevant environmental and occupational questionnaires, which are more likely to identify a potential inciting agent when compared with clinical history [23]. Several proposed questionnaires were designed for ILD, but the majority included only a limited list of main exposures, and all of them lacked validation. The more comprehensive and HP-focused is the evidence-based screening questionnaire for suspected exposures proposed by Petnak et al. [26] that should be adapted to the geographical area and local working practices in order to maximize its efficiency.
- Involvement of industrial hygienists in order to use their expertise to inspect the building systems, collect samples, and identify potential exposures other than the obvious ones [8, 27].
- Raising awareness about the possible impact of a specific exposure in an occupational setting among workers from a potentially hazardous environment. They could attribute acute respiratory symptoms associated with fever and malaise not only to an acute respiratory infection but also could suspect HP as a potential alternative diagnosis and seek earlier for a medical opinion.

#### 5.1.2. Serum-Specific IgG

Measuring specific serum IgG against the offending antigen is a diagnostic tool used in patients with ILD that can distinguish HP with a sensitivity and specificity of 83 and 68%, respectively [27]. In a large study by Samson et al., patients with elevated specific serum IgG levels had a nearly 10-fold increased likelihood of subsequent HP diagnosis [28]. Most laboratories have developed panels for HP, which usually include common serum-specific IgG, while specialized centers extract antigens for testing from the patient's environment [29] and thus reveal hidden exposures, which give more accurate results [30], but still lack validation. However, it should be noted that positive circulating antibodies do not prove causality [27]. In most cases, they are just markers of exposure because many asymptomatic individuals show similar levels of humoral responses [3]. This is why positive serum-specific IgG should be interpreted carefully, mostly in clinical and imaging-suggestive cases, thus avoiding potential false-positive results.

#### 5.1.3. Antigen Inhalation Challenge Tests

Following inhaling a nebulized solution containing the suspected antigen, clinical assessment, laboratory tests, lung function, and imaging results are analyzed. The response criteria are extensive and typically assessed at 8–12 hours after provocation. They include respiratory symptoms, increased clinical and laboratory signs of inflammation (fever, C reactive protein, and leukocytosis), and decreased blood oxygenation and lung function. The procedure is not standardized and lacks validation, but several studies show a sensitivity and specificity that ranges from 73% and 84%, respectively, to 100% [31, 32]. Being a challenge test, it possesses a risk of a severe reaction; therefore, it should be assigned only when other investigations have been uninformative and need to be performed in specialized centers. Neither of the guidelines currently recommends the antigen inhalation challenge tests [6, 8].

### 5.2. Bronchoalveolar Lavage

Although not unanimously recognized as a valuable tool for diagnosing HP by the guidelines (Table 3) [6, 8], the bronchoalveolar lavage (BAL) fluid provides not only evidence of a T-cell activation triggered by a certain antigen exposure but can also be used to rule out an alternative diagnosis, such as an infection. Flow cytometry may be helpful to support a diagnosis of HP whenever the CD4+ to CD8+ ratio has low values ranging between 0.5 and 1.5. In contrast, higher ratios suggest pulmonary sarcoidosis, another granulomatous disorder with lymphocytosis in the BAL. In limited cases, especially in smoking patients, screening for CD1a+ T- cell numbers in BAL fluid could help distinguish an initial stage of Langerhans cell histiocytosis from HP. However, due to high variability, flow cytometry of the BAL has limited clinical utility [33].

It is widely accepted that a high percentage of alveolar lymphocytosis increases the likelihood of HP. Thus, a lymphocyte count >30% in the BAL in an patient with ILD may increase the diagnostic confidence for HP to highly probable and make the lung biopsy unnecessary [6, 8]. However, the lack of BAL lymphocytosis in the fibrotic type does not exclude it [25], while the absence of lymphocytosis in the nonfibrotic pattern almost rules out the possibility of HP [6]. BAL lymphocytosis may also have a prognostic role suggesting more inflammation or less fibrosis, becoming a predictor of treatment response [34].

From a clinical point of view, for diagnostic purposes, BAL has the highest utility in ILDs, especially in cases with suggestive symptoms and positive exposure history but with an indeterminate HRCT pattern.

### 5.3. Chest Imaging

Chest HRCT is a centerpiece investigation for the diagnosis of HP. When suspected, two images should be acquired: one after deep inspiration and the second after prolonged expiration [8]. Typically, there are signs of parenchymal lesions, mainly of the interstitial space, featured on HRCT by ground-glass opacities and mosaic attenuation, and of small airways involvement suggested by the presence of ill-defined centrilobular nodules and air-trapping (Figure 1) [6]. These features can be found in both fibrotic and nonfibrotic HP. Another almost pathognomonic imaging sign that has a specificity of 93% for a diagnosis of fibrotic HP [35] is the “three density sign,” previously known as the “headcheese sign,” which resembles a patchwork of lung lobules with normal density, alternating with lobules with ground-glass attenuation and lobules with decreased density and decreased vessel size due to air-trapping [36]. For the fibrotic pattern of HP, there is a coexistence of previously described opacities with traction bronchiectasis and honeycombing, which are most likely not to show a preferable distribution as it was previously stated [37], since some recent studies found only 10% of fibrotic HP cases having an upper lobe predominance [38, 39].

**Figure 2:**
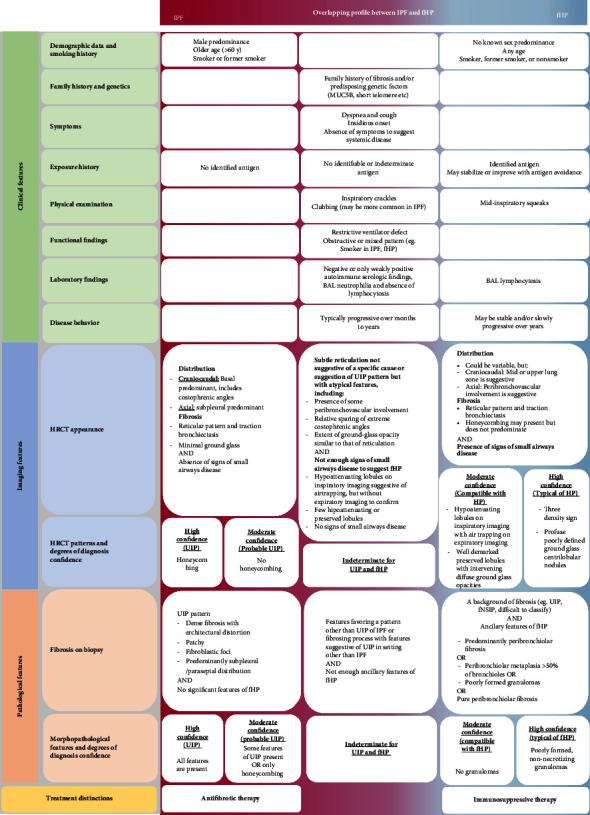
An approach to the assessment of clinical, imaging, and pathological features in patients with IPF, fHP, or both as primary diagnostic considerations in the absence of alternative causes (adapted after Marinescu et al. [40]).

Existing guidelines describe specific features of single clinical entities. At the same time, in real life, clinicians face a real challenge in distinguishing an ILD from another, with the most significant difficulties found in fibrotic ILDs.  Figure 2 proposes an approach when IPF and fibrotic HP are major diagnostic considerations emphasizing the clinical, imaging, and pathological distinctions.

### 5.4. Lung Function

Pulmonary function tests (PFTs) are part of the mandatory assessment of a patient with HP, as they are for any ILD. While unable to describe any specific changes, PFTs are a valuable tool in assessing the severity, predicting the outcome, and following up for progression. In HP, regardless of the phenotype, the main ventilatory abnormality is restriction; therefore, a reduced forced vital capacity (FVC) is a common finding, followed by a low carbon monoxide diffusing capacity (DLCO) as a marker of involvement of the interstitial space [6]. Reduction in these two parameters is a strong indicator of progressive pulmonary fibrosis, which can be established in cases of >10% decrease in FVC alone or 5–9% decline in FVC, >15% reduction in DLCO with deteriorating symptoms, and/or progression on CT scan over 6–12 months despite treatment [41]. More comprehensive studies could reveal the unique feature of small airway involvement in HP, especially in the fibrotic type. They usually demonstrate air-trapping, expressed by increased residual volume (RV) and increased residual volume and total lung capacity ratio (RV/TLC), as was shown by Dias and colleagues. [42]. Earlier studies also found predominant obstructive abnormalities, suggesting the presence of bronchiolitis and emphysema [43]. While changes in PFTs stated above can be described in both phenotypes at baseline, with treatment and antigen avoidance in the nonfibrotic HP, lung function could be completely recovered. In the fibrotic type, only modest improvements can be achieved.

The six-minute walking test (6MWT), a cheap, easy-to-perform investigation, is a composite of exercise tolerance, the degree of pulmonary vasculopathy, gas exchange efficacy, and patient mobility [44], that can be used as a follow-up and a prognostic tool. Although unable to demonstrate a distinctive pattern for HP, shorter walking distances and higher levels of oxygen desaturation (SaO_2_) are associated with severe disease and poor outcome [45]. Among the few studies performed specifically on HP patients, a recent paper showed that longer walking distance indicates a good response to treatment. Moreover, reductions in the 6MWT distance correlate with DLCO, while higher levels of desaturation were associated with lower vital capacity (VC) and DLCO values [46].

### 5.5. Lung Biopsy

In most cases suspected of HP, obtaining a lung tissue sample is unnecessary, and the diagnosis relies on the clinical features, a positive exposure history, and a suggestive imaging pattern. However, in inconclusive cases, after an MDT discussion, a lung biopsy could be recommended. There are several approaches for performing a lung biopsy: transbronchial forceps biopsy, transbronchial lung cryo-biopsy (TBLC), or surgical lung biopsy (SLB). The optimal method selection is usually determined by the HP phenotype, comorbidities, functional status of the patient, and procedure-related complications risks, such as the potential to induce an acute exacerbation, bleeding, pneumothorax, and last but not least—local experience in performing the procedure. Despite a low diagnostic yield of only 37% (95% CI 32–42) [6], the ATS/JRS/ALAT guideline recommends transbronchial forceps biopsy in cases suggestive of nonfibrotic HP instead of more invasive methods of obtaining lung tissue, while in fibrotic HP, the latter are preferred [6] (Table 3). TBLC has a higher estimated diagnostic yield for HP (82%) [47], although it possesses an increased risk of bleeding and pneumothorax [48]. With similar potential complications as TBLC but with an additional increased risk of postprocedural exacerbations and death, having a diagnostic yield of 96%, SLB remains the gold standard for tissue sampling [6].

As expected, HP's imaging phenotypes are reflected by a range of histopathological patterns.

The non-fibrotic HP can be morphologically represented by a triad of lesions affecting both the interstitium and the small airways, typically involving predominantly the central regions of the secondary pulmonary lobule, being expressed by lymphocyte inflammatory infiltrate like in cellular non-specific interstitial pneumonia (NSIP) with focal organizing pneumonia, cellular bronchiolitis, and poorly or loosely formed granulomas. All these patterns may be accompanied by scattered multinucleated giant cells in various compartments of the lung parenchyma, predominantly in the peribronchiolar interstitium. Other common features of HP are the presence of cytoplasmatic inclusions such as cholesterol clefts, Schaumann bodies, or asteroid bodies [49, 50].

The fibrotic HP is morphologically represented by the same background of interstitial pneumonia and bronchiolitis with over-imposed fibrosis, having an important distinction from other fibrotic ILDs given by the bronchiolocentric distribution of both inflammation and fibrosis, accompanied by the presence of granulomas or multinucleated giant cells [51]. Occasionally, features of fibrotic NSIP and fibroblastic foci, the hallmark of UIP, can be detected [52].

## 6. Prognostic Factors

The course of HP can be influenced by a large number of factors: demographic data, antigen exposure, chronicity of disease, smoking status, comorbidities, genetics, and some clinical data.

Older age is widely reported to be associated with increased mortality. Fernández Pérez and colleagues found that patients older than 65 have significantly higher mortality rates than younger ones (115.9 vs. 37.5 per 1000 person-years) [11]. The same database recorded a worse survival in male patients, although females registered a higher prevalence of HP [11]. Similar studies from Spain and China have found no difference in mortality between sexes [53, 53, 54, 54], whereas a Portuguese cohort recently proposed the ILD-GAP index as a good predictor for mortality in fibrotic HP [55].

Interestingly, while commonly the smoking effect on the lung is injurious, in the case of two granulomatous diseases, such as sarcoidosis and hypersensitivity pneumonitis, smoking may be associated with a decrease in the incidence of disease [56]. However, although older reports found that acute HP is less common in smokers, patients who smoke are more prone to develop lung fibrosis [57]; thus, smoking is associated with worse overall survival in patients with HP.

HP involves an environmental antigen; therefore, avoiding the antigen is the key to disease resolution. Since in half of the cases, the antigen cannot be identified [5], avoiding further exposure becomes impossible. There are conflicting data on the impact of antigen exposure on disease course in patients with HP. Complete antigen avoidance resulted in no recurrence or development of fibrosis in patients with nonfibrotic HP and longer survival in both fibrotic and nonfibrotic HP [10, 58]. Still, despite complete antigen avoidance, patients with fibrotic HP developed progressive lung fibrosis [58].

Currently, in most cases, discrimination between nonfibrotic and fibrotic HP can be made with confidence based on HRCT features. The fibrotic pattern (UIP) found at imaging, or the histopathological investigation poses an increased mortality risk [59]. According to Salisbury et al., HP patients who displayed honeycombing at HRCT showed a similar survival rate to IPF patients [60]. Moreover, honeycombing in patients with HP defines progressive fibrosis, which is associated with a higher mortality rate [61]. Similar findings in terms of prognosis were detected when the UIP pattern was determined in lung biopsy samples [6]. Conversely, ground-glass opacification, air-trapping, and mosaic attenuation on HRCT have been associated with improved survival [62].

Certain circulating biomarkers, such as KL-6, YKL-40, and CCL17, or markers of autoimmunity (positive ANA and autoimmune thyroiditis), have been associated with disease progression [22]. Due to the intensified release by the regenerating type II pneumocytes in the affected lung, specifically, KL-6 is noticeably raised in ILDs with a strong inflammatory background. Considering nonfibrotic HP and fibrotic HP as two ends of the inflammation-fibrosis spectrum, KL-6 has the potential to differentiate these forms of HP; therefore, it can be used as a prognostic tool as well as an instrument able to discriminate fibrotic HP from IPF [63, 64]. Since most studies regarding serum biomarkers derive from Asian countries, these biomarkers may need validation in other populations. Additionally, short telomeres and reduced BAL lymphocytosis may be linked to an inability to respond to immunosuppressive treatment [65].

About half of fibrotic HP cases develop pulmonary hypertension, and this complication is directly associated with the disease severity and also with worse survival [66]. Higher mortality rates are also found in HP patients with lower FVC and DLCO values [53, 54]. Moreover, patients who develop progressive pulmonary fibrosis tend to show an IPF-like behavior with similar mortality rates [41].

Despite the multitude of HP prognostic factors, their impact on an individual patient remains unknown. Each patient should be considered as a unit, and a holistic approach, including the impact of comorbidities, will improve prognosis evaluation and quality of life. Interestingly, a recent study identified three clusters with distinct comorbidities that could represent different phenotypes in HP. The authors claimed that mortality and respiratory hospitalizations were higher in the cluster dominated by cardiovascular diseases [67].

## 7. Treatment

Currently, there is no unanimous agreement regarding the therapeutic approach of HP. Since this ILD entity has a predominant inflammatory character driven by exposure to an inciting antigen, antigen avoidance and corticosteroids/immunosuppressive drugs are the mainstay of HP treatment. At the same time, antifibrotic agents show promising results in the progressive fibrotic phenotype of HP. Another treatment option designated for advanced disease is the lung transplant.

As previously stated, identification and complete antigen avoidance, although somewhat challenging, are key to a better outcome in patients with HP [10]. Especially in an occupational setting, hygienist interventions would increase the effectiveness of the measures for antigen detection and its elimination from the environment [68].

Although there is limited evidence supporting this therapeutic approach, the two types of HP benefit from slightly different treatment options.

In nonfibrotic HP, corticosteroids are often the drugs of choice, and commonly, the treatment regimen consists of prednisone 0.5–1 mg/kg/day for 1-2 weeks, followed by a gradual tapering until a maintenance dose of 10 mg/day [69]. There are no current guidelines that would state otherwise, but, in clinical practice, if there is a radiological, functional, and clinical improvement and the patient has ceased the exposure, the corticosteroids could be tapered off after a period of several months. Data suggest lung function improvement in short-term follow-up studies, while the long-term treatment does not show any favorable effect [70].

In the case of fibrotic HP, treatment recommendations are more equivocal. The empirical initial dose will be maintained for 4–8 weeks and gradually tapered to the lowest efficient dose, usually 10 mg/day. Clinical, imaging, and functional data will dictate treatment duration and dose, but given the inflammatory background of the disease and the fact that the inciting antigen remains undiscovered in around half of cases, immunosuppression may be required for an extended period (months, years).

When more prolonged use of corticosteroids is required due to progression and/or frequent relapses or when antigen avoidance is not possible, earlier transition to steroid-sparing agents, such as mycophenolate (MMF) or azathioprine (AZA), might be a reasonable alternative, with fewer adverse either in monotherapy or in combination with low dose steroids [71]. In the case of fibrotic HP, compared to corticosteroids, AZA/MMF was associated with similar mortality risk [71], despite improved lung function after one year, fewer adverse events, and better adherence to immunosuppressive treatment [72].

There is growing evidence about the benefits of antifibrotic therapy in patients with fibrotic HP that show pulmonary fibrosis progression despite adequate treatment. Various combinations of worsening of respiratory symptoms, decline of FVC and DLCO, and/or evidence of increasing fibrosis on HRCT are used to define progression. However, despite a recently published guideline [73], there is still a lot of confusion regarding the timing of the antifibrotic therapy initiation, which antifibrotic agent is preferred, whether it should be as an add-on therapy to immunosuppression or alone, issues that have been addressed extensively elsewhere [74]. Currently, only nintedanib has been approved for use in fibrotic HP in several countries [75], while pirfenidone has been studied only in small cohorts with promising results [76–79].

## 8. Prevention

Avoiding exposure to the offending antigen plays a central role in preventing HP; this is why regular workplace inspection for potential sources of antigens is crucial. Since most antigens are either of bacterial or fungal origin, using antimicrobial and antifungal solutions, cleaning, removing water-damaged objects, disinfecting, and sterilizing the equipment have proved to be efficient in reducing the antigen load in the environment. Personal protective equipment such as respirators and masks are able to limit inhalation of the inciting antigens, while dust respirators have limited efficiency in protecting against organic matter [22].

## 9. Questions for Future Research

Despite the advances that have been made lately, there are still knowledge gaps, which impose future research about HP. There is a compelling necessity for standardized and validated diagnostic tools (exposure questionnaires, isolation of the antigen from the patient's environment, serum-specific IgG panels, challenge test standardization, and BAL lymphocytosis threshold). Artificial intelligence shows promising results and has demonstrated an enormous potential that could facilitate the diagnostic process and be a valuable tool for research. While treatment of nonfibrotic HP is unequivocal, managing fibrotic HP is still challenging, even for experienced clinicians. Due to the low level of evidence for current immunosuppressive treatment and concerning long-term outcomes, better quality trials are warranted for longer follow-up periods. Similarly, there is a need for better evidence about the potential benefit of antifibrotics in the progressive fibrotic HP phenotype.

More studies about proteomics and genotyping of this category of patients would give a better overview of HP pathogenesis, which will enable the identification of biomarkers for predicting disease behavior.

## Figures and Tables

**Table 1:** List of jobs with possible antigen exposure sources.

Occupation area	Jobs with occupational exposures	Possible sources of antigens	Antigen name
Agriculture	Farmers	Decaying vegetation (hay and grass) and soil (farms with animals, such as cattle and horses)	*Absidia corymbifera, Lichtheimia corymbifera,* and *Saccharopolyspora rectivirgula*
		Plant surfaces, fruit, honeybee's environment, animal, or human feces (soil with organic fertilizer)	*Pantoea agglomerans* and *Streptomyces albus*
		Moldy hay, straw, plant material, seeds (sunflower, wheat, rye, barley, maize, in-shell peanuts, pecans, and peas), beans (mung, soybeans, soy products, and green coffee beans); cereals (corn, rice, and wheat); dry substrates (straw and seeds); air in agricultural and human-associated environments	Thermophilic actinomycetes (*Saccharopolyspora rectivirgula, Thermoactinomyces vulgaris,* and *Thermoactinomyces sacchari), Pantoea agglomerans Wallemia sebi, Wallemia mellicola,* and *Wallemia muriae*
		Compost	*Streptomyces thermohygroscopicus, Thermoactinomyces vulgaris,* and *Saccharomonospora viridis*
		Peat moss	*Saccharomonospora viridis* (thermoactinomyces *viridis)* and *Aspergillus* spp. (e.g., *A. fumigatus* and *A. niger*)
	Onion growers	Onion peels	*Penicillium* spp., Aspergillus flavus, and Aureo pullulans
	Bagasse workers	Bagasse, hay, straw, and moldy plants	*Aerobacter cloacae, Thermoactinomyces sacchari,* and *Thermoactinomyces vulgaris*
	Mushroom growers	Contaminated compost, edible mushroom manure, hay, and dusty soil	*Streptomyces albus* and *Streptomyces thermohygroscopicus*

Poultry	Bird breeders (hen workers, pigeon breeders, turkey, duck, goose handlers, and feather pluckers)	Parakeet, pigeon, chicken, turkey, goose, and duck proteins	Bird droppings or feathers

Food industry	Cheese producers	Cheese processing and washing	*Penicillium notatum, Penicillium viridicutum, Penicillium roqeforti, Penicillium verrucosum, Penicillium casei, Aspergillus fumigatus, Aspergillus niger,* and *Aspergillus pullulans*
	Bakers	Contaminated flour and aspergillus enzyme in baking agents	*Aspergillus fumigatus*
	Soy sauce brewers	Fermentation starter for soy sauce	*Aspergillus oryzae*
	Salami factory workers	Dried sausage dust white coating on salami	*Penicillium glabrum, Penicillium* spp., and *Aspergillus fumigatus*
	Wine growers	Mold on grapes	*Botrytis cinerea*

Constructions	Plasterers, insulators, and varnishers	Glue, polyurethane foam, paint, plastic, resins, and varnishes	Isocyanate acid anhydrides, toluene diisocyanate, methylene diphenyl, isocyanate, and hexamethylene diisocyanate
	Woodworkers and wood trimmers	Oak, cedar, pine, spruce, mahogany dust, and contaminated wood trimmings	*Alternaria* spp*, Bacillus subtilis Rhizopus* spp, and *Mucor* spp

Miscellaneous	Wind instruments players	Trombone, trumpet, flute, saxophone, and clarinet	*Mycobacteria chelonae* or other mycobacteria species *Fusarium* spp and *Phoma* spp
	Air conditioner/humidifier/swamp cooler	Contaminated water	*Thermoactinomyces* spp., *Aspergillus* spp., *Penicillium* spp. *Aureobasidium* spp*, Candida albicans*, and *Thermophilic actinomycetes*
	Dental technicians	Dental products	Methyl acrylates
	Lifeguards, pool workers	Contaminated water jets and sprays	*Pseudomonas* spp.
	CPAP machine, nebulizers	Contaminated water	*Rhodotorula minuta* and *Candida* spp
	Sawmill workers	Maple bark	*Cryptostroma corticale*

**Table 2:** Clinical presentation of HP^*∗*^.

	Antigen exposure	Onset of symptoms	Symptoms	Physical examination	Outcome	Prognostic
Non-fibrotic HP	High-level intermittent exposure of usually an identifiable antigen	Hours or days following significant exposure	Acute or insidious onset of productive cough, dyspnea, and fatigue often associated with an intermittent flu-like syndrome (fever, chills, malaise, cough, chest tightness, dyspnea, and headache)	Diffuse fine bibasilar crackles, mid-inspiratory squeaks	Symptoms resolve gradually within 12 hours to several days after exposure removal and may recur following re-exposure	In case of exposure' avoidance may have a favorable prognosis with the possibility of stabilization or complete recovery
Fibrotic HP	Low-level continuous exposure to a frequently unknown antigen	Months after exposure	Insidious disease with no apparent acute episodes. Progressive dyspnea, cough, fatigue, malaise, and/or weight loss	Bilateral crackles, cyanosis, *cor pulmonale*, and finger clubbing (in 50% of patients)	End-stage fibrotic disease. Exacerbation may occur despite antigen avoidance	Poor prognosis

^*∗*^Adapted from Nogueira et al. [3].

**Table 3:** Face-to-face recommendations on the same diagnostic tools given by the guidelines [6, 8].

Diagnostic tools	Chest guideline	ATS/JRS/ALAT guideline
History of exposure	Thorough clinical history of exposures	Thorough history to identify potential exposures ± questionnaires
	Role of occupational medicine specialist and an environmental hygienist	—
	Clinical improvement after exposure avoidance—support diagnosis	—
	Serum-specific IgG, IgA testing	Serum-specific IgG testing

HRCT role	HRCT integrated with clinical findings	HRCT essential role

BAL	Not routinely recommended	BAL for lymphocyte count recommended for non-fibrotic HP and suggested for fibrotic HP (adding TBB increases the diagnostic yield)

Lung biopsy	Indicated when clinical, laboratory, HRCT, and BAL do not yield the diagnosis	TBB for non-fibrotic HP
	Integrating biopsy with clinical and HRCT	TBLC for fibrotic HP
	No recommendation regarding the preferred methods	SLB suggested only after alternative diagnostic options have been exhausted

MDT	For deciding the need for lung biopsy	For deciding the need for TBLC or SLB
	For diagnostic decision-making	For diagnostic decision-making

BAL: bronchoalveolar lavage, HRCT: high-resolution computer tomography, MDT: multidisciplinary team, TBB: transbronchial biopsy, TBLC: transbronchial cryo-biopsy, and SLB: surgical lung biopsy.

## Data Availability

No underlying data were collected or produced in this study.

## References

[1] Vasakova M., Morell F., Walsh S., Leslie K., Raghu G. (2017). Hypersensitivity pneumonitis: perspectives in diagnosis and management. *American Journal of Respiratory and Critical Care Medicine*.

[2] Campbell J. M. (1932). Acute symptoms following work with hay. *BMJ*.

[3] Nogueira R., Melo N., Novais e Bastos H. (2019). Hypersensitivity pneumonitis: antigen diversity and disease implications. *Pulmonology*.

[4] Sullivan A., Shrestha P., Lanham T., Lanham E., Baba M. (2020). Bird Fancier’s lung: an underdiagnosed etiology of dyspnea. *Respiratory Medicine Case Reports*.

[5] Quirce S., Vandenplas O., Campo P. (2016). Occupational hypersensitivity pneumonitis: an EAACI position paper. *Allergy*.

[6] Raghu G., Remy-Jardin M., Ryerson C. J. (2020). Diagnosis of hypersensitivity pneumonitis in adults. An official ATS/JRS/ALAT clinical practice guideline. *American Journal of Respiratory and Critical Care Medicine*.

[7] Selman M., Pardo A., King T. E. (2012). Hypersensitivity pneumonitis: insights in diagnosis and pathobiology. *American Journal of Respiratory and Critical Care Medicine*.

[8] Fernandez Perez E. R., Travis W. D., Lynch D. A. (2021). Executive summary: diagnosis and evaluation of hypersensitivity pneumonitis: CHEST guideline and expert panel report. *Chest*.

[9] Villar A., Ojanguren I., Muñoz X., Cruz M. J., Morell F. (2016). Hypersensitivity pneumonitis: challenges in diagnosis and management, avoiding surgical lung biopsy. *Seminars in Respiratory and Critical Care Medicine*.

[10] Fernandez Perez E. R., Swigris J. J., Forssen A. V. (2013). Identifying an inciting antigen is associated with improved survival in patients with chronic hypersensitivity pneumonitis. *Chest*.

[11] Fernández Pérez E. R., Kong A. M., Raimundo K., Koelsch T. L., Kulkarni R., Cole A. L. (2018). Epidemiology of hypersensitivity pneumonitis among an insured population in the United States: a claims-based cohort analysis. *Annals of the American Thoracic Society*.

[12] Karakatsani A., Papakosta D., Rapti A. (2009). Epidemiology of interstitial lung diseases in Greece. *Respiratory Medicine*.

[13] Kornum J. B., Christensen S., Grijota M. (2008). The incidence of interstitial lung disease 1995-2005: a Danish nationwide population-based study. *BMC Pulmonary Medicine*.

[14] Barber C. M., Wiggans R. E., Carder M., Agius R. (2017). Epidemiology of occupational hypersensitivity pneumonitis; reports from the SWORD scheme in the UK from 1996 to 2015. *Occupational and Environmental Medicine*.

[15] Rittig A. H., Hilberg O., Ibsen R., Lokke A. (2019). Incidence, comorbidity and survival rate of hypersensitivity pneumonitis: a national population-based study. *ERJ Open Research*.

[16] Blanc P. D., Annesi-Maesano I., Balmes J. R. (2019). The occupational burden of nonmalignant respiratory diseases. An official American thoracic society and European respiratory society statement. *American Journal of Respiratory and Critical Care Medicine*.

[17] Pereira C. A., Gimenez A., Kuranishi L., Storrer K. (2016). Chronic hypersensitivity pneumonitis. *Journal of Asthma and Allergy*.

[18] Fishwick D. (2012). New occupational and environmental causes of asthma and extrinsic allergic alveolitis. *Clinics in Chest Medicine*.

[19] Vandenplas O. (2011). Occupational asthma: etiologies and risk factors. *Allergy, Asthma & Immunology Research*.

[20] Lhoumeau A., Pernot J., Georges M. (2012). Hypersensitivity pneumonitis due to isocyanate exposure in an airbag welder. *European Respiratory Review*.

[21] Rooijackers J. M., Zaat V., Veltkamp M. (2020). Home environment exposure assessment in hypersensitivity pneumonitis. *European Respiratory Journal*.

[22] Costabel U., Miyazaki Y., Pardo A. (2020). Hypersensitivity pneumonitis. *Nature Reviews Disease Primers*.

[23] Morell F., Villar A., Montero M. A. (2013). Chronic hypersensitivity pneumonitis in patients diagnosed with idiopathic pulmonary fibrosis: a prospective case-cohort study. *The Lancet Respiratory Medicine*.

[24] Salisbury M. L., Myers J. L., Belloli E. A., Kazerooni E. A., Martinez F. J., Flaherty K. R. (2017). Diagnosis and treatment of fibrotic hypersensitivity pneumonia. Where we stand and where we need to go. *American Journal of Respiratory and Critical Care Medicine*.

[25] Fernandez Perez E. R., Koelsch T. L., Leone P. M., Groshong S. D., Lynch D. A., Brown K. K. (2020). Clinical decision-making in hypersensitivity pneumonitis: diagnosis and management. *Seminars in Respiratory and Critical Care Medicine*.

[26] Petnak T., Moua T. (2020). Exposure assessment in hypersensitivity pneumonitis: a comprehensive review and proposed screening questionnaire. *ERJ Open Research*.

[27] Johannson K. A., Barnes H., Bellanger A. P. (2020). Exposure assessment tools for hypersensitivity pneumonitis. An official American thoracic society workshop report. *Annals of the American Thoracic Society*.

[28] Samson M. H., Vestergaard J. M., Knudsen C. S., Kolstad H. A. (2021). Serum levels of IgG antibodies against Aspergillus fumigatus and the risk of hypersensitivity pneumonitis and other interstitial lung diseases. *Scandinavian Journal of Clinical and Laboratory Investigation*.

[29] Bellanger A. P., Reboux G., Rouzet A. (2019). Hypersensitivity pneumonitis: a new strategy for serodiagnosis and environmental surveys. *Respiratory Medicine*.

[30] Sterclova M., Kremlackova V., Mottlova V., Bruzova M., Sojka P., Vasakova M. (2021). Quantitative assessment of specific serum IgGs may verify source of environmental exposure in extrinsic allergic alveolitis (EAA). *Cogent Medicine*.

[31] Munoz X., Sanchez-Ortiz M., Torres F., Villar A., Morell F., Cruz M. J. (2014). Diagnostic yield of specific inhalation challenge in hypersensitivity pneumonitis. *European Respiratory Journal*.

[32] Ishizuka M., Miyazaki Y., Tateishi T., Tsutsui T., Tsuchiya K., Inase N. (2015). Validation of inhalation provocation test in chronic bird-related hypersensitivity pneumonitis and new prediction score. *Annals of the American Thoracic Society*.

[33] Meyer K. C., Raghu G. (2011). Bronchoalveolar lavage for the evaluation of interstitial lung disease: is it clinically useful?. *European Respiratory Journal*.

[34] De Sadeleer L. J., Hermans F., De Dycker E. (2020). Impact of BAL lymphocytosis and presence of honeycombing on corticosteroid treatment effect in fibrotic hypersensitivity pneumonitis: a retrospective cohort study. *European Respiratory Journal*.

[35] Walsh S. L. F., Richeldi L. (2019). Demystifying fibrotic hypersensitivity pneumonitis diagnosis: it’s all about shades of grey. *European Respiratory Journal*.

[36] Hamblin M., Prosch H., Vasakova M. (2022). Diagnosis, course and management of hypersensitivity pneumonitis. *European Respiratory Review*.

[37] Silva C. I., Muller N. L., Lynch D. A. (2008). Chronic hypersensitivity pneumonitis: differentiation from idiopathic pulmonary fibrosis and nonspecific interstitial pneumonia by using thin-section CT. *Radiology*.

[38] Chung J. H., Montner S. M., Adegunsoye A. (2017). CT findings associated with survival in chronic hypersensitivity pneumonitis. *European Radiology*.

[39] Salisbury M. L., Gross B. H., Chughtai A. (2018). Development and validation of a radiological diagnosis model for hypersensitivity pneumonitis. *European Respiratory Journal*.

[40] Marinescu D. C., Raghu G., Remy-Jardin M. (2022). Integration and application of clinical practice guidelines for the diagnosis of idiopathic pulmonary fibrosis and fibrotic hypersensitivity pneumonitis. *Chest*.

[41] Cottin V. (2023). Criteria for progressive pulmonary fibrosis: getting the horse ready for the cart. *American Journal of Respiratory and Critical Care Medicine*.

[42] Dias O. M., Baldi B. G., Ferreira J. G. (2018). Mechanisms of exercise limitation in patients with chronic hypersensitivity pneumonitis. *ERJ Open Research*.

[43] Lalancette M., Carrier G., Laviolette M. (1993). Farmer’s lung. Long-term outcome and lack of predictive value of bronchoalveolar lavage fibrosing factors. *American Review of Respiratory Disease*.

[44] Harari S., Wells A. U., Wuyts W. A. (2022). The 6-min walk test as a primary end-point in interstitial lung disease. *European Respiratory Review*.

[45] Caminati A., Bianchi A., Cassandro R., Rosa Mirenda M., Harari S. (2009). Walking distance on 6-MWT is a prognostic factor in idiopathic pulmonary fibrosis. *Respiratory Medicine*.

[46] Lewandowska K. B., Sobiecka M., Boros P. W. (2023). New 6-minute-walking test parameter-distance/desaturation index (DDI) correctly diagnoses short-term response to immunomodulatory therapy in hypersensitivity pneumonitis. *Diagnostics*.

[47] Chami H. A., Diaz-Mendoza J., Chua A. (2021). Transbronchial biopsy and cryobiopsy in the diagnosis of hypersensitivity pneumonitis among patients with interstitial lung disease. *Annals of the American Thoracic Society*.

[48] Troy L. K., Grainge C., Corte T. J. (2020). Diagnostic accuracy of transbronchial lung cryobiopsy for interstitial lung disease diagnosis (COLDICE): a prospective, comparative study. *The Lancet Respiratory Medicine*.

[49] Kitaichi M., Shimizu S., Tamaya M., Takaki M., Inoue Y., Sharma O. P. (2013). Pathology of hypersensitivity pneumonitis. *Clinical Focus Series, Hypersensitivity Pneumonitis*.

[50] Castonguay M. C., Ryu J. H., Yi E. S., Tazelaar H. D. (2015). Granulomas and giant cells in hypersensitivity pneumonitis. *Human Pathology*.

[51] Churg A., Muller N. L., Flint J., Wright J. L. (2006). Chronic hypersensitivity pneumonitis. *The American Journal of Surgical Pathology*.

[52] Gaxiola M., Buendia-Roldan I., Mejia M. (2011). Morphologic diversity of chronic pigeon breeder’s disease: clinical features and survival. *Respiratory Medicine*.

[53] Ojanguren I., Morell F., Ramon M. A. (2019). Long-term outcomes in chronic hypersensitivity pneumonitis. *Allergy*.

[54] Wang L. J., Cai H. R., Xiao Y. L., Wang Y., Cao M. S. (2019). Clinical characteristics and outcomes of hypersensitivity pneumonitis: a population-based study in China. *Chinese Medical Journal*.

[55] Mendonça Almeida L., Fernandes A. L., Gouveia Cardoso C. (2022). Mortality risk prediction with ILD-GAP index in a fibrotic hypersensitivity pneumonitis cohort. *Therapeutic Advances in Respiratory Disease*.

[56] Murin S., Bilello K. S., Matthay R. (2000). Other smoking-affected pulmonary diseases. *Clinics in Chest Medicine*.

[57] Warren C. P. (1977). Extrinsic allergic alveolitis: a disease commoner in non-smokers. *Thorax*.

[58] Nishida T., Kawate E., Ishiguro T., Kanauchi T., Shimizu Y., Takayanagi N. (2022). Antigen avoidance and outcome of nonfibrotic and fibrotic hypersensitivity pneumonitis. *ERJ Open Research*.

[59] Akashi T., Takemura T., Ando N. (2009). Histopathologic analysis of sixteen autopsy cases of chronic hypersensitivity pneumonitis and comparison with idiopathic pulmonary fibrosis/usual interstitial pneumonia. *American Journal of Clinical Pathology*.

[60] Salisbury M. L., Gu T., Murray S. (2019). Hypersensitivity pneumonitis: radiologic phenotypes are associated with distinct survival time and pulmonary function trajectory. *Chest*.

[61] Selman M., Pardo A., Wells A. U. (2023). Usual interstitial pneumonia as a stand-alone diagnostic entity: the case for a paradigm shift?. *The Lancet Respiratory Medicine*.

[62] Chung J. H., Zhan X., Cao M. (2017). Presence of air trapping and mosaic attenuation on chest computed tomography predicts survival in chronic hypersensitivity pneumonitis. *Annals of the American Thoracic Society*.

[63] d’Alessandro M., Bergantini L., Cameli P. (2020). Krebs von den Lungen-6 as a biomarker for disease severity assessment in interstitial lung disease: a comprehensive review. *Biomarkers in Medicine*.

[64] Sanchez-Diez S., Munoz X., Ojanguren I. (2022). YKL-40 and KL-6 levels in serum and sputum of patients diagnosed with hypersensitivity pneumonitis. *Journal of Allergy and Clinical Immunology: In Practice*.

[65] Adegunsoye A., Morisset J., Newton C. A. (2021). Leukocyte telomere length and mycophenolate therapy in chronic hypersensitivity pneumonitis. *European Respiratory Journal*.

[66] Oliveira R. K. F., Ota-Arakaki J. S., Gomes P. S. (2018). Pulmonary haemodynamics and mortality in chronic hypersensitivity pneumonitis. *European Respiratory Journal*.

[67] Prior T. S., Wälscher J., Gross B., Bendstrup E., Kreuter M. (2022). Clusters of comorbidities in fibrotic hypersensitivity pneumonitis. *Respiratory Research*.

[68] Kawamoto Y., Oda S., Tanaka M. (2021). Antigen avoidance in people with hypersensitivity pneumonitis: a scoping review. *Heart & Lung*.

[69] Spagnolo P., Rossi G., Cavazza A. (2015). Hypersensitivity pneumonitis: a comprehensive review. *Journal of Investigational Allergology & Clinical Immunology*.

[70] Cano-Jiménez E., Rubal D., Pérez de Llano L. A. (2017). Farmer’s lung disease: analysis of 75 cases. *Medicina Clínica*.

[71] Adegunsoye A., Oldham J. M., Fernandez Perez E. R. (2017). Outcomes of immunosuppressive therapy in chronic hypersensitivity pneumonitis. *ERJ Open Research*.

[72] Morisset J., Johannson K. A., Vittinghoff E. (2017). Use of mycophenolate mofetil or azathioprine for the management of chronic hypersensitivity pneumonitis. *Chest*.

[73] Raghu G., Remy-Jardin M., Richeldi L. (2022). Idiopathic pulmonary fibrosis (an update) and progressive pulmonary fibrosis in adults: an official ATS/ERS/JRS/ALAT clinical practice guideline. *American Journal of Respiratory and Critical Care Medicine*.

[74] Rajan S. K., Cottin V., Dhar R. (2023). Progressive pulmonary fibrosis: an expert group consensus statement. *European Respiratory Journal*.

[75] Flaherty K. R., Wells A. U., Cottin V. (2019). Nintedanib in progressive fibrosing interstitial lung diseases. *New England Journal of Medicine*.

[76] Shibata S., Furusawa H., Inase N. (2018). Pirfenidone in chronic hypersensitivity pneumonitis: a real-life experience. *Sarcoidosis Vasculitis and Diffuse Lung Diseases*.

[77] Mateos-Toledo H., Mejia-Avila M., Rodriguez-Barreto O. (2020). An open-label study with Pirfenidone on chronic hypersensitivity pneumonitis. *Archivos de Bronconeumología*.

[78] Tzilas V., Tzouvelekis A., Bouros E. (2020). Clinical experience with antifibrotics in fibrotic hypersensitivity pneumonitis: a 3-year real-life observational study. *ERJ Open Research*.

[79] Behr J., Prasse A., Kreuter M. (2021). Pirfenidone in patients with progressive fibrotic interstitial lung diseases other than idiopathic pulmonary fibrosis (RELIEF): a double-blind, randomised, placebo-controlled, phase 2b trial. *The Lancet Respiratory Medicine*.

